# Use of pulse oximetry during initial assessments of children under five with pneumonia: a retrospective cross-sectional study from 14 hospitals in Ethiopia

**DOI:** 10.29392/joghr.3.e2019016

**Published:** 2019-02-01

**Authors:** Habtamu Seyoum Tolla, Mekitew Letebo, Yigeremu Abebe Asemere, Alemayehu Berhanu Belete, Tegegn Chote Tumbule, Zinabie Feleke Fekadu, Dinkineh Bikila Woyessa, Simret Ameha, Yibeltal Mekonen Feyisa, Felix Lam

**Affiliations:** 1 Clinton Health Access Initiative, Addis Ababa, Ethiopia; 2 Ethiopia Federal Ministry of Health, Addis Ababa, Ethiopia; 3 Clinton Health Access Initiative, Boston, Massachusetts, USA

## Abstract

**Background:**

Hypoxemia, a fatal condition characterized by low concentration of oxygen in the blood, is strongly associated with death among children with pneumonia. Ethiopia’s Federal Ministry of Health launched its first National Oxygen and Pulse Oximetry Scale-up road map to improve access and utilization of pulse oximetry and oxygen. This study aimed to describe the use of pulse oximetry during the initial patient assessment among children under five diagnosed with pneumonia and serves as a benchmark to measure progress of the road map.

**Methods:**

The study design was an observational study using retrospective review of patient medical records at 14 hospitals. Medical records of 443 children age 0-59 months with a primary diagnosis of pneumonia were randomly selected for review. The primary outcome was whether an arterial blood oxygen saturation (SpO_2_) measurement was recorded in the patient’s medical record at the initial assessment.

**Results:**

Overall, 10% (95% confidence interval CI = 4%-22%) of patient medical records had a SpO_2_ measurement. Admitted patients were more likely to have a SpO_2_ measurement recorded in their medical records than patients treated in the outpatient department (*P*<0.01). Among admitted patients, 19% (95% CI = 8%-38%) had a SpO_2_ measurement compared to 3% (95% CI = 1%-11%) of patients treated in the outpatient department.

**Conclusion:**

In Ethiopia, patients under five with a primary diagnosis of pneumonia are rarely screened for hypoxemia with a pulse oximeter, and hypoxemia may be severely underdiagnosed. Much needs to be done to improve the routine use of pulse oximetry.

Pneumonia is a leading cause of mortality among children 0-59 months in Ethiopia (*1*). In 2016, it was estimated that 30,000 children 0-59 months died from pneumonia in Ethiopia, making it the fifth highest figure in the world (*2*). Hypoxemia, a fatal condition characterized by an abnormally low concentration of oxygen in the blood, is strongly associated with death among children with pneumonia and is estimated to affect 20% of pneumonia cases in Africa (*3*-*5*). To detect hypoxemia, the World Health Organization (WHO) recommends using pulse oximetry, a non-invasive technology that measures oxygen saturation of the hemoglobin in the blood (*6**,* *7*). Compared to clinical signs and symptoms alone, pulse oximetry can correctly identify 20-30% more cases of hypoxemia in children (*8*-*11*). Furthermore, a systematic review found that use of pulse oximetry may lower mortality rates, reduce length of hospital stay, and change physicians’ decisions on diagnosis and treatment (*12*).

Despite evidence of the benefits of pulse oximetry, there is low adoption of pulse oximetry in low resource settings, especially where there has been no intervention to introduce oximeters. In Kenya, three out of 22 hospitals had pulse oximeters in 2012 (*13*). In Malawi, a study found three out of five hospitals had pulse oximeters in the pediatric inpatient wards though none of the departments routinely screened for hypoxemia during admissions (*14*). Prior to an intervention to improve oxygen delivery in twelve Nigerian hospitals, only 3% of children and neonates had oximetry documented on admission (*15*).

In Ethiopia, national guidelines, such as the Integrated Management of Newborn and Childhood Illness (IMNCI) guidelines and the Pediatric Care Manual for Hospitals, advise healthcare workers to use pulse oximetry to assess patients for hypoxemia. However, there is no local evidence on whether pulse oximetry is being used for detection of hypoxemia. A situational analysis did find that only 45% of pediatric inpatient departments in hospitals had functional pulse oximeters. To expand the use of pulse oximetry and medical oxygen, the Ethiopian Federal Ministry of Health (FMOH) developed the *Medical Oxygen and Pulse Oximetry Scale Up Road Map in Ethiopia* that provides a multi-year plan for comprehensively rolling out oxygen equipment and pulse oximeters across the public health system (*16*, *17*). We conducted this study to provide estimates on pulse oximetry utilization, which can serve as an early benchmark for measuring the country’s progress towards achieving the goals set forth in the road map.

## Objectives

The primary objective of this study was to quantify the use of pulse oximetry during the initial patient assessment (e.g., at triage, the initial evaluation, or both). The secondary objective of the study was to investigate differences in pulse oximetry use by the patient’s sex, age category (e.g., 0-11 months, 12-23 months, etc.), and hospital department (e.g., outpatient or inpatient). The study focused on pediatric patients between 0-59 months of age and with a diagnosis of pneumonia.

## METHODS

### Study design

The study design was an observational study using retrospective review of patient medical records. The study randomly selected patient medical records from fourteen hospitals for review and extracted key study variables.

### Study setting

The study was conducted in fourteen hospitals located in five regions of Ethiopia: Addis Ababa City Administration, Amhara, Oromia, Southern Nations, Nationalities, and Peoples Region (SNNPR), and Tigray. These regions constitute approximately 90% of the country’s total population (*18*). The hospitals included referral, general, and primary levels. The primary hospital is part of the Primary Healthcare Unit system that includes health catchment centers and health posts and is expected to service populations of 60,000 to 100,000. The general hospital is the next level of care and serves 1-1.5 million people while the tertiary hospital is a specialized system serving 3.5-5 million people.

### Study participants

The study population was children 0-59 months of age with a diagnosis of pneumonia and had been treated at a hospital between February 1, 2016 and April 30, 2017. We first randomly selected one referral, one general, and one primary hospital from each of the regions (except in Addis Ababa where there are no primary hospitals). Data collectors visited the selected hospitals and reviewed the patient registers at the outpatient and inpatient departments. For each eligible pediatric patient, the data collectors recorded their information in MS Excel (Microsoft Inc, Seattle WA, USA); the child’s medical record number, name, sex, department from which the patient was seen, and date of visit. A separate patient list was created for each study facility. After listing all pediatric patients meeting these criteria, the data collectors used systematic random sampling to select medical records for review using a sampling interval (i.e. total participants in facility divided by required sample size). To this end, the data collectors first sorted the list by the patient’s date of visit and randomly selected a number between one and the sampling interval. Children were then sampled according to the sampling interval, and this process continued until the sample size for the facility was met. Once the sampling was complete, the data collectors worked with the facility staff to locate the patient medical records that were selected using the child’s medical record number.

### Outcomes

The primary outcome of the study was documentation of an arterial blood oxygen saturation measurement (SpO_2_) at triage, the initial evaluation, or both.

### Data sources and study procedures

The data collection team consisted of three secondary degree holders who are familiar with clinical services and documentation practices in Ethiopian hospitals. Data collection was conducted between May 3, 2017 and June 16, 2017. Data collectors first introduced themselves to the facility head. After introducing themselves and the study, the data collectors requested the time of a facility staff member to help them locate the registers and selected medical records. Once the registers were located, the data collectors completed the listing form and conducted the sampling procedure. For the medical records, a standardized data abstraction form was used to gather data on the key variables. The data collection tool and procedures were piloted at St. Peter Hospital in Addis Ababa and further refined before being used for the study.

### Sample size

The sample size was calculated to provide an estimate for the number of medical records required for review. The primary indicator for conducting the sample size was the proportion of medical records of children 0-59 months with a diagnosis of pneumonia that had a documented SpO_2_ reading. To be as precise as possible in our sample size estimate, we also applied a finite population correction factor to the sample size calculation. The finite population correction factor, rather than assuming an infinite population, accounts for the number of under five pneumonia cases treated at the 14 study facilities during the study period. The sample size calculation used was:

$$n☒\frac{N\left[\frac{Z^{☒}p\left(☒☒p\right)}{e^{☒}}\right]}{\left[\frac{Z^{☒}p\left(☒☒p\right)}{e^{☒}}\right]☒N☒☒}$$

where n is the sample size required for the study, N is the total population of children 0-59 months diagnosed with pneumonia seen at the study facilities during the study period, z is 1.96 (i.e. the Z-score for a 95% confidence level), P is the population proportion of children 0-59 months with pneumonia and a SpO_2_ reading, and e is the absolute precision margin. For our study, we used 0.05 for the precision margin. Since there are no available estimates for the proportion of children 0-59 months with pneumonia and a SpO_2_ reading, we used 0.5 to obtain the most conservative sample size estimate. For N, we used the number of children 0-59 months of age found in the pediatric outpatient and inpatient registers with a diagnosis of pneumonia. There were 15,224 children 0-59 months with pneumonia who were listed in the registers for the study period. Using our sample size equation above, we calculated a sample size of 375 medical records of children 0-59 months with a diagnosis of pneumonia is required for the study. To account for potential inability to find the medical records at the facility, we increased our sample size by 20%. Thus, our final sample size for the study was 450 medical records of children 0-59 months with a diagnosis of pneumonia.

We distributed the sample size among the 14 hospitals by allocating a proportion that was equal to the facility’s share of the total number of pneumonia cases seen during the study period. Similar to the finite population correction, we used the number of children 0-59 months of age with a diagnosis of pneumonia found in the patient registers during the study period.

### Statistical analysis

An experienced data entry clerk electronically entered the data from the standardized abstraction forms using Epi Info 7 (Centers for Disease Control and Prevention, Atlanta GA, USA). Data analysis was conducted using Stata 14 (StataCorp, College Station TX, USA). Descriptive statistics were carried out to estimate the proportion of pediatric patient’s age 0-59 months with a diagnosis of pneumonia that had a SpO_2_ measurement recorded. We estimated cluster standard errors using the facility as the sampling cluster. Two sided one-sample t-tests were conducted to test for differences in SpO_2_ recording by patient’s sex, age, and the department where the patient was treated. Variables with a *P*-value less than 0.05 were considered statistically significant. The reporting of this study conforms to STROBE recommendations.

### Ethics approval

The study was reviewed and approved by the Ethiopian Public Health Institute (Ref. No. CHAI/CS/925/18).

### Role of the funding source

The funder of the study had no role in the study design, data collection, data analysis, data interpretation, or writing of the report. The corresponding author had full access to all data and had final responsibility for the decision to submit for publication.

## RESULTS

### Participants

**Table 1** presents the number of children 0-59 months diagnosed with pneumonia at each facility during the study period, the sample size allocated to each facility, and the total number of patient medical records found. The study found 15,224 children 0-59 months with a diagnosis of pneumonia listed in the patient registers between February 1, 2016 and April 30, 2017. From that list, the study selected 450 medical records for review. Of the 450 patient medical records randomly selected for review, 7 (2%) could not be located.

**Table 1:** Summary of sampling results

REGION	FACILITY	LEVEL	NO. OF CHILDREN 0-59 MONTHS WITH PNEUMONIA LISTED IN PATIENT REGISTERS BETWEEN FEB 1, 2016 AND APR 30, 2017	SAMPLE SIZE CALCULATION	SAMPLE SIZE ACHIEVED
Addis Ababa	Yekatit 12 Hospital	Referral	744	22	22
	Tirunesh Beijing Hospital	General	406	12	12
Amhara	Felegehiwot Hospital	Referral	847	25	25
	Woldia Hospital	General	845	25	25
	Ataye Hospital	Primary	509	15	15
Oromia	Shashamane Hospital	Referral	1,083	32	32
	Bishoftu Hospital	General	1,861	55	54
	Batu Hospital	Primary	1,658	49	48
SNNPR	Hawassa Referral Hospital	Referral	505	15	15
	Adare Hospital	General	2,470	73	72
	Leku Hosital	Primary	1,489	44	43
Tigray	Ayder Hospital	Referral	744	22	22
	Mekelle Hospital	General	1,590	47	45
	Hawzen Hospital	Primary	473	14	13
Total			15,224	450	443

SNNPR – Southern Nations, Nationalities, and People’s Region

**Table 2** presents characteristics of the sample. 53% (95% CI = 47%-58%) of the medical records reviewed were of male patients and 47% (95% CI = 42%-53%) were of female patients. Children less than 1 year made up 42% (95% CI = 37%-46%) of the medical records reviewed. Of the entire study sample, 56% (95% CI = 50%-61%) were treated in the outpatient department and 44% (95% CI = 39%-50%) were admitted to pediatric wards.

**Table 2:** Characteristics of patient medical records

SELECTED CHARACTERISTICS	N	%	95% CI
Sex:			
Male	234	53	47-58
Female	209	47	42-53
Age:			
0-11 months	183	42	37-46
12-23 months	115	26	22-31
24-35 months	46	11	8-14
36-47 months	50	11	8-15
48-59 months	46	11	7-15
Department:			
Outpatient	246	56	50-61
Inpatient	196	44	39-50

CI – confidence interval

### SpO_2_ measurement documented in patient medical records

**Table 3** presents the proportion of medical records with a SpO_2_ measurement recorded. Overall, 10% (95% CI = 4%-22%) of patient medical records had a SpO_2_ measurement. There were no differences in the proportion of medical records with a SpO_2_ measurement by sex or age group. However, admitted patients were more likely to have a SpO_2_ measurement recorded in their medical records than patients treated in the outpatient department (*P*<0.01). Among admitted patients, 19% (95% CI = 8%-38%) had a SpO_2_ measurement compared to 3% (95% CI = 1%- 11%) for patients treated in the outpatient department.

**Table 3:** Percent of patient medical records with SpO_2_ measurement recorded

SELECTED CHARACTERISTICS	%	95% CI	*P*-VALUE
Total	10.2	4.4-21.8	N/A
Sex:			
Male	9.8	4.2-21.4	Ref
Female	10.5	4.0-25.0	0.83
Age:			
0-11 months	14.2	6.1-29.6	Ref
12-23 months	7.0	2.8-16.5	0.04
24-35 months	15.2	6.3-32.3	0.68
36-47 months	6.0	1.6-20.3	0.07
48-59 months	2.2	0.2-18.4	0.05
Department:			
Outpatient	2.8	0.7-10.6	Ref
Inpatient	18.9	8.1-38.0	0.00
Facility:			
Yekatit 12 Hospital	22.7	*	*
Tirunesh Beijing Hospital	83.3	*	*
Felegehiwot Hospital	4.0	*	*
Woldia Hospital	0.0	*	*
Ataye Hospital	0.0	*	*
Shashamane Hospital	0.0	*	*
Bishoftu Hospital	0.0	*	*
Batu Hospital	0.0	*	*
Hawassa Referral Hospital	53.3	*	*
Adare Hospital	11.1	*	*
Leku Hosital	0.0	*	*
Ayder Hospital	9.1	*	*
Mekelle Hospital	24.4	*	*
Hawzen Hospital	0.0	*	*

SpO_2_ – arterial blood oxygen saturation, CI – confidence interval

* Not estimable due to clustering at the facility.

Recording of SPO_2_ measurement varied substantially among facilities. In Tirunesh Beijing Hospital in Addis Ababa, 83% of patient medical records had a SpO_2_ measurement. This was the highest among the 14 study facilities. The next highest proportions were 53% and 24% in Hawassa Referral Hospital and Mekelle Hospital, respectively. In seven facilities, none of the patient medical records reviewed had a SpO_2_ measurement.

Among patients with a SpO_2_ measurement recorded, 57% (95% CI = 30%-80%) had a measurement that was less than 90% (**Table 4**).

**Table 4:** Percent of patient medical records with SpO_2_ measurements <90%

SPO_2_<90%	N	%	95% CI
No	19	43.2	20.0-69.8
Yes	25	56.8	30.2-80.0

SpO_2_ – arterial blood oxygen saturation, CI – confidence interval

## DISCUSSION

To our knowledge, this is the first study to provide estimates on SpO_2_ measurement recording in Ethiopia across multiple hospital settings. Use of pulse oximetry to screen for hypoxemia in children 0-59 months with pneumonia presenting to Ethiopian hospitals is low. The low proportion of patient medical records with a SpO_2_ measurement documented likely reflects the low availability of pulse oximeters and low awareness of healthcare workers to routinely use pulse oximeters during triaging and diagnosing of patients. When a SpO_2_ measurement was recorded, we found that over half of the patients had hypoxemia. These findings suggest that many patients with hypoxemia may be missed. While clinical signs and symptoms may be able to identify some of these hypoxemic patients, 20-30% of cases may be missed if assessed with clinical signs or symptoms alone (*8*-*10*). Given the large burden of pneumonia in Ethiopia and the potentially large proportion of pneumonia cases that present with hypoxemia, increasing access and utilization of pulse oximeters and oxygen will be critical in reducing pneumonia mortality.

We also found that pulse oximetry is more often used among inpatient pneumonia cases than outpatient cases. This likely reflects differences in availability of pulse oximeters in the respective departments. However even among inpatient pneumonia cases, which are likely more severe, pulse oximetry use was low. Notably, Tirunesh Beijing Hospital had a high proportion of patients with SpO_2_ documented. A case study on Tirunesh Beijing Hospital may help other facilities replicate its success.

Ethiopia’s road map calls for scale-up of pulse oximetry at all levels of the health system. This study only included hospitals as the situational analysis in the road map found that no health centers had any pulse oximeters. However, improving pulse oximetry utilization at all levels will require more than making pulse oximeters available at the facilities. A survey of healthcare workers in Cambodia found that in addition to supply, other barriers included lack of policies, guidelines, training, and perceived high costs (*19*). Another mixed-methods evaluation study in 12 hospitals of Southwest Nigeria found rapid uptake of pulse oximetry from 3% of admitted pediatric patients to 95%, but uptake was variable across the facilities (*15*). The study suggested that key qualities that facilitated rapid uptake were leadership at the facilities, an institutional culture to encourage and reinforce new behaviors, and the compatibility of pulse oximetry with the healthcare worker’s motivation and activities.

Continued measurement of pulse oximetry utilization will be an important metric of success for Ethiopia’s road map. However, there is very little routine data available on pulse oximetry utilization despite recognition of its importance and embracement of SpO_2_ as a “fifth vital sign”. The lack of available data on pulse oximetry use was one of the impetuses for this study. It is likely feasible to have routine measurement of pulse oximetry utilization as other disease areas already have metrics that measure use of diagnostics. For malaria, another major contributor of childhood mortality in Ethiopia, lab-confirmed malaria is routinely tracked against clinically diagnosed malaria in the country’s health management information system (HMIS) summary forms to measure progress on use of blood diagnostic tests for confirming malaria cases. In HIV, the number of women attending an antenatal care visit that are tested is routinely captured as well. Incorporating pulse oximetry utilization into routine measurement systems may be important to increasing its uptake.

### Limitations

The study used retrospective reviews of patient medical records to measure pulse oximetry utilization. However in resource-limited settings, patient medical records may not fully reflect clinical practice. Studies in low resource settings have found incomplete and low quality information being captured in medical records (*20*-*22*). Healthcare workers in Ethiopia and other low resource countries face high patient loads so patient documents may be incomplete. This may bias our results downwards if healthcare workers are conducting pulse oximetry but not recording the measurements. Thus, our results may be an underestimate. Additionally, patient records may become lost, thus introducing another source of bias in our results. However in our study, we were only unable to locate 2% of the patient files selected for review, thus this source of bias is likely minimal.

The sampling design of the study is also limited to hospital settings and children under five with a diagnosis of pneumonia. We limited the sampling frame to only hospitals in Addis Ababa, Amhara, Oromia, SNNPR, and Tigray and randomly selected the hospitals. The results are likely representative of hospitals in these regions. However, hospitals can be quite heterogeneous in terms of patient load, staffing, and resources as seen with a few hospitals in our sample, and therefore, future studies should consider sampling a more exhaustive number of hospitals. The study does not include any health centers or health posts, however, pulse oximeters at these facility types are non-existent.

Our population was also limited to children under five with a diagnosis of pneumonia that were seen in the pediatric outpatient or inpatient departments. It is unlikely that children of other age groups or diagnosed with other conditions would be more or less likely to be assessed with pulse oximetry though it is possible. For example the strong association between pneumonia and hypoxemia may lead healthcare workers to assess children with pneumonia for hypoxemia more frequently than children diagnosed with other conditions. Given our sampling design, the results of the study are not generalizable to children in other age groups, diagnosed with other conditions, or managed elsewhere in the facility (eg, intensive care unit). However taking the results of this study in context with results on the availability of pulse oximeters from the National Oxygen and Pulse Oximetry Scale-up road map, we are confident that use of pulse oximetry is generally low throughout Ethiopia.

The study was also not powered to explore differences in pulse oximetry utilization. While we present some results by patient age, sex, and facility department, the low sample size prevented further analyses to explore differences among regions, facilities, facility levels, and other patient characteristics. Additionally, practices within facilities were likely similar. Our analysis accounted for clustering at the facility which resulted in conservative estimates for our standard errors and further hindered our ability to conduct exploratory analyses. Furthermore, the study was not powered to evaluate the health impact from pulse oximetry utilization. This would be a strong area for further study, and we are aware of a stepped-wedge trial underway in Southwest Nigeria that examines the impact of pulse oximetry and oxygen system strengthening on patient outcomes (*23*).

## CONCLUSIONS

Despite existing guidelines recommending routine use of pulse oximetry, there is low utilization of pulse oximetry during the initial assessment and admissions for pediatric pneumonia cases in Ethiopian hospitals.

## Data sharing statement

The dataset and analysis are available at Seyoum, Habtamu; Letebo, Mekitew; Asemere, Yigeremu; Belete, Alemayehu; Tumbule, Tegegn; Fekadu, Zinabie; Woyessa, Dinkineh; Ameha, Simret; Feyisa, Yibeltal; Lam, Felix, 2019, “Replication Data for: Use of pulse oximetry during initial assessments of children under five with pneumonia: a retrospective cross-sectional study from 14 hospitals in Ethiopia”, https://doi.org/10.7910/DVN/AVGPJ9, Harvard Dataverse.
